# Is Team Sport the Key to Getting Everybody Active, Every Day? A Systematic Review of Physical Activity Interventions Aimed at Increasing Girls' Participation in Team Sport

**DOI:** 10.3934/publichealth.2017.2.202

**Published:** 2017-05-11

**Authors:** Rosalie Allison, Emma L. Bird, Stuart McClean

**Affiliations:** 1. Public Health England, Gloucester, GL1 3NN, UK; 2. Faculty of Health and Life Sciences, University of the West of England, Bristol, BS16 1QY, UK

**Keywords:** team sport, health promotion, physical activity, systematic review, adolescent girls, gender inequality

## Abstract

**Background:**

It is estimated that 21% of boys and 16% of girls in England meet recommended physical activity guidelines. Team sport has the potential to increase physical activity levels; however, studies show that gender-based factors can influence girls' participation in team sport. Furthermore, evidence for the effectiveness of interventions promoting team sport among girls is limited. This systematic review aimed to assess the impact of physical activity interventions on secondary school-aged girls' (aged 11-18 years) participation in team sport and to identify potential strategies for increasing participation.

**Methods:**

Electronic databases and grey literature were systematically searched for studies of interventions targeting team sport participation among girls in the UK. Results were exported to Refworks, duplicates removed and eligible studies identified. Extracted data included: participant details, such as sample size and age; components of the intervention; outcomes assessed; and each study was quality appraised. Due to heterogeneity across studies, results were presented narratively.

**Results:**

Four studies sourced from the grey literature met the inclusion criteria. Findings suggest that physical activity interventions can encourage girls to try new sports, but evidence is limited in relation to sustained participation. Potential strategies for promoting participation included: consultation with girls, implementation of appropriate peer-leaders and friendship group strategies, early intervention and consideration of intervention setting.

**Conclusions:**

This review highlights the limited availability of evidence on the effectiveness of physical activity interventions for promoting team sport participation among girls in the UK. Findings indicate that future research is needed to improve the methodological quality of complex intervention evaluation. Physical activity interventions may have the potential to encourage girls to try team sport, but their impact on sustained participation, and subsequent physical activity outcomes, is less apparent.

## 1. Introduction

Globally, physical inactivity is now the fourth leading cause of death [1]. Physical inactivity is an independent risk factor for premature death and diseases such as coronary heart disease, stroke, some cancers, type 2 diabetes, osteoporosis, and depression, and it is a major contributor to obesity in adults [2]. Because these conditions have not historically been prevalent among children and adolescents, attention to physical inactivity as a health risk among young people has been slower to emerge. Evidence is accumulating, however, that the onset of many chronic diseases of adulthood lies in childhood [3]. Moreover, recent epidemiological data from the US shows dramatic increases among youth in the incidence of type 2 diabetes, obesity [4], high blood pressure, dyslipidemia, and sleep disorders [5].

Recommended guidelines state that children and adolescents need to accumulate at least 60 minutes of moderate-to-vigorous physical activity (MVPA) on most days of the week [6]. In England, subjective data suggest that only 21% of boys and 16% of girls in the 5–15 years age group, meet these recommended guidelines [7]. Not only are youths not meeting recommended physical activity levels, but gender differences are present. Globally, girls are less active than boys [8] and there is a more pronounced decline in physical activity during adolescence in girls than in boys [9]. By age 14, girls are dropping out of sports at two times the rate of boys [10].

To date, much of the research on physical inactivity has focused on the barriers to participation, especially in girls [11]. Existing systematic reviews explore the role of social support [12] and the effectiveness of after-school interventions [13] but few studies have tested interventions designed specifically for girls, and very few have shown positive intervention effects on physical activity [14]. In the last decade, there has been a gradual increase in the number of randomised controlled trials that have evaluated the impact of physical activity-based interventions on adolescent girls [15], but minimal evaluations of UK-based interventions.

UK based interventions for young girls' participation in team sport are likely to be introduced in an educational context, therefore there is a need to identify the qualities gained from team sport that are related to the national curricula and the context of individual development. Evidence suggests that team sport may have the potential to provide greater mental and physical health benefits than other forms of physical activity, and participation in team sport has been linked with better mental health, more resilience to the stresses of modern living, increased life satisfaction [16], higher grades at school, and lower risk-taking behaviours such as substance abuse [17]. Internationally there has been increased interest in using sport as a medium for developing 'life skills; in learners. “Life skills” can be described as: those skills that enable learners to succeed in the different environments in which they live, such as: school, home, their future workplace and society. These life skills can be broken down into: behavioural (communicating effectively with peers and adults); cognitive (making effective decisions); interpersonal (being assertive), or intrapersonal (setting goals). Additional benefits, derived from developing life skills through participation in team sport, include: the ability to operate in a team, deal more effectively with emotions and the aspects of winning or losing; and in dealing with discipline and routine [17].

There is still uncertainty about the causal relationship between sport participation and the processes that may lead to improved life prospects. One theory is that “It is probably not the mere participation in sport that enhances positive development but the individual's experience in sport that may be the critical factor” [18]. Therefore, literature suggests that team sport could, not only increase physical activity levels, but play an important part in improving the life prospects of young people, and is therefore an appropriate and unique focus for this systematic review.

In order to develop and implement targeted strategies to increase team sport participation and, consequently, physical activity levels among adolescent girls, there is a need to understand trends in, and influences on, adolescent girls' participation in team sport. Although previous systematic reviews have examined the effectiveness of interventions designed to increase physical activity in general [19],[20], to date there is no known systematic review investigating the effectiveness of physical activity interventions that aim to promote participation in team sport specifically. As such, this systematic review addressed the following question: What is the evidence for the effectiveness of team sport interventions aimed at secondary school aged girls (aged 11–18 years) on team sport participation and wider physical activity outcomes?

## 2. Materials and Methods

Following the PRISMA guidelines [21], a three-part search strategy was used to identify studies meeting the inclusion criteria. Firstly, articles for consideration in the review were located by searching the following electronic databases: CINAHL Plus; AMED — The Allied and Complementary Medicine Database; British Education Index; MEDLINE; PsycINFO; SPORTDiscus; and Child Development & Adolescent Studies. EBSCohost Research Databases was the Interface used. The keywords used referred to the participant (adolescent girls), intervention and outcome (team sport) variables of interest. Specifically, the search strategy involved the following search terms: (girl* OR school girl* OR female* OR young wom* OR adolescen* OR teen* OR child* OR pre-adolescen* OR pre-teen* OR high school* OR “year 7 or 8”) AND (intervention* OR campaign* OR strateg* OR promot* OR program* OR initiativ*) AND (physical activit* OR exercis* OR sport* OR game* OR basketball* OR volleyball* OR water polo* OR handball* OR lacrosse* OR cricket* OR football* OR netball* OR hockey* OR team sport* OR team ball sport* OR team game*). See supplementary 1 for the full search strategy used to search the electronic databases.

Next, grey literature was searched for unpublished/working documents, in order to minimise publication bias. The approach taken in this systematic review involved searching the national sports council's websites (e.g. Sport England), and sifting through their archives, in turn, identifying any evaluations of interventions (See Supplementary 2 for full details of the grey literature searches). From this, and from searching the reference lists of potentially relevant studies, a number of key authors became apparent. Emails were sent to a nine of these in an attempt to gain some advice on which journals to search for published evaluations of interventions.

Once all sources had been systematically searched, the list of citations was exported to Refworks in order to identify eligible studies. By scanning the title, and abstract if necessary, studies that did not fit the inclusion criteria were removed. When the title and abstract did not provide sufficient information the full manuscript was examined. Inclusion criteria included: (a) studies of interventions aimed at or including secondary school girls (aged 11–18 years); (b) studies evaluating physical activity interventions with a team sport element. In this review team sport interventions included any activity in which a group of individuals, on the same team, work together to accomplish an ultimate goal, which is usually to win. This can be done in a number of ways, such as: outscoring the opposing team. Examples of team sports include: basketball, football, netball, and hockey, amongst others; (c) studies that report, either in full or including, change in participation in team sport following an intervention e.g. netball, football, rugby, hockey etc. For the purposes of this review, “participation” was defined as attendance at, at least one team sport session; (d) studies based in the UK; (e) studies carried out in the last 10 years (2005–2015); (f) any study that contained the desired outcomes. Study design was not restricted as different methodological approaches could contribute to understanding the variations in outcomes of physical activity interventions in relation to participation in team sport and could therefore be relevant to this systematic review. For example, qualitative data could illuminate why a particular intervention or approach had variable impacts and suggest ways of dealing with this, and quantitative data could indicate its relative effectiveness in relation to levels of participation in team sport, overall [22]. Studies were excluded if they: did not report female-specific outcomes; focused on general physical activity interventions and did not report on specific team sport outcomes; did not meet all of the inclusion criteria.

An 11-item critical appraisal tool adapted from existing tools (CASP, UK [23], NICE [24], The University of Auckland [25], and the Centre for Evidence-based Medicine, University of Oxford [26]) was developed (see Supplementary 3 for the critical appraisal template). The following rating system was employed: “++” when all or most of the checklist criteria had been fulfilled, where they have not been fulfilled the conclusions were very unlikely to alter; “+” when some of the checklist criteria have been fulfilled, where they have not been fulfilled, or not adequately described, the conclusions were unlikely to alter; “−” when few or no checklist criteria had been fulfilled and the conclusions were likely or very likely to alter. “NA” was reserved for those study design aspects that were not applicable given the study design under review. An additional section was added to the critical appraisal tool for criteria that were rated “+” or “−”. This section referred to whether there was a reference to the evaluation not being for academic purposes, or the weaknesses in description of methods being acknowledged. If there was reference to this, and the paper was “++” quality in the rest of the study design, it was used in data synthesis, but noted that the findings from these studies could only be used as a guide or suggestions for future research, as it could not be confirmed that the conclusions were unbiased.

Data were extracted on: study design; population details including sample size, country and recruitment method; full details of the intervention; all study design outcomes; relevant results; author's conclusions; recommendation for the future (see supplementary 4 for the data extraction template). As this systematic review brought together themes and results from a relatively heterogeneous set of studies, meta-analysis was not deemed appropriate. Instead, all data were narratively synthesised, an approach commonly used for synthesis of diverse, but not well developed evidence [27]–[29].

## 3. Results

### 3.1. Study Selection

**Figure 1.**
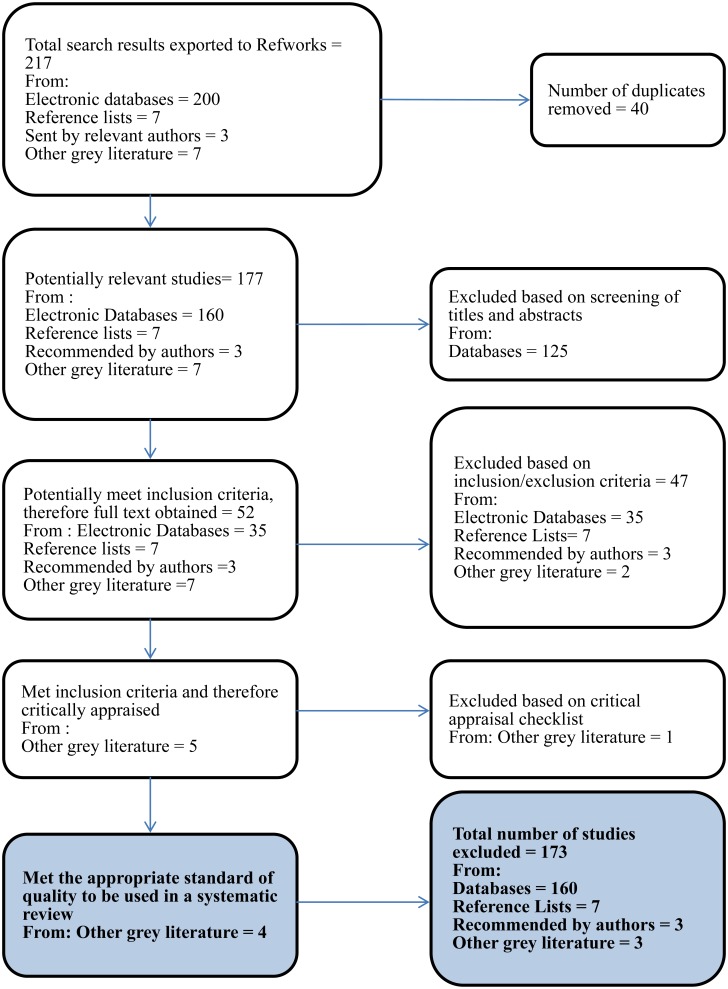
PRISMA Flowchart of search results

The electronic database search produced 200 papers, a further seven papers were identified from reference lists, three suggested by contacted authors, and a further seven from grey literature sources. After removing duplicates (n = 40), 177 publications remained. After titles and abstracts were examined 52 full-text papers were read and assessed further for eligibility. Of those, five articles were identified as suitable by meeting all of the inclusion criteria. After critical appraisal, it was decided that four studies would be included in the systematic review as one study was excluded due to limited explanation of methods and therefore conclusions could not be justified (Figure 1).

### 3.2. Study Characteristics

As shown in Table 1, two of the interventions selected were based in Scotland [30],[31] and the remaining two in England [32],[33]. Three of the studies utilised quantitative methodologies, such as questionnaires and attendance registers [30]–[33]; all studies included a qualitative component, such as interviews and focus groups [30]–[33].

(1) Study population

Three interventions were offered solely to females [30]–[32], whereas “Sportivate” [33] had a mixed gender focus, yet reported in detail on gender specific participation, retention and most popular sports.

**Table 1.** Summary of included studies, including information on the participants

Intervention	Aim	Location and setting	Data Collection Methods	Critical Appraisal Score^1^
Fit for Girls Evaluation [30] **(Fit for Girls)**	To promote physical activity among girls aged 11–16 years. It is specifically aimed at girls who are least likely to be active, who are not currently active in the extra-curricular setting and who may opt out of curricular physical education (PE).	11–16 year old females, Scotland, school-based setting	Quantitative: a national survey of a cohort of girls pre and post-intervention an online survey of PE staff and Active Schools coordinators in years two and three of the programme Qualitative: focus group discussions with ‘disengaged’^2^ girls in four case study schools focus group discussions with PE staff in four case study schools in-depth interviews with local and national stakeholders.	10/11
Us Girls: Engaging Young Women from Disadvantaged Communities in Sport [32] **(Us Girls)**	Us Girls was designed to get 30,000 16–25 year old young women from disadvantaged communities more active, by providing them with fitness and sport opportunities within their local communities.	16–25 year old females, England, community-based setting	The research included interviews and focus groups with project managers, coaches, and participants.	9/10
Sportivate Programme Evaluation [33] **(Sportivate)**	To increase participation of both male and female participants aged 11 to 25 year olds who may not seek out sporting opportunities themselves, would not prioritise doing sport in their own time or those who are doing sport for a very limited amount of time.	11–25 year old, mixed gender, England, mixed settings	An online data portal to gather ‘real time’ data including project information, registration forms and attendance registers A hardcopy exit postcard survey to reveal the intentions of young people to continue to take part in sport An online survey to track levels of participation by young people three months after taking part in the Sportivate programme. A review process to provide qualitative feedback regarding their progress with the programme A review process for project providers or deliverers to provide qualitative feedback about the successes and challenges experienced.	10/10
Evaluation of “Girls on the Move” Programme: Summary Report [31] **(Girls on the Move)**	The programme was designed to increase the physical activity levels of girls and young women in Scotland by addressing the barriers that prevent them from participating in physical activity.	11–18 year old females, Scotland, community setting	Quantitative: surveys attendance sheets Qualitative: observation interviews group discussions	9/11

^1^ Each of the criteria was awarded 1 point if they were rated “++” (when all or most of the checklist criteria had been fulfilled, where they have not been fulfilled the conclusions were very unlikely to alter) or “+” (when some of the checklist criteria have been fulfilled, where they have not been fulfilled, or not adequately described, the conclusions were unlikely to alter ). If they were ‘NA’ (reserved for those study design aspects that were not applicable given the study design under review), this criteria was not included in the overall score.

^2^ Adolescent girls with low levels of overall physical activity and who are not engaging with any physical activity offered to them. These adolescent girls are often identified as low active, hard to reach or disengaged.

(2) Description of the Interventions

The different approaches utilised to achieve the differing aims of the interventions can be found in Table 2. All approaches were unique and no specific component was utilised in all interventions.

**Table 2.** Components of the Individual Interventions

Name of Intervention	a) Training	b) Action Plan	c) Grant	d) Partnership Working	e) Resources	f) How to Guide	g) Gender specific research and insights	h) Merchandise
Fit for Girls [30]	X	X	X	X	X			
Us Girls [32]	X			X	X	X	X	X
Sportivate [33]				X				
Girls on the Move [31]			X					

**(a) Training**

Two of the interventions specified that training or workshops for staff were involved in their intervention [30],[32]. In “Fit for Girls” [30], the fact that the trainer was a PE teacher themselves was seen as a particular strength, as it facilitated a mutual understanding between the attendees and the trainer. Flexibility in intervention timing, length and location of training workshops was noted in both interventions as crucial to the success of both projects. For example, for some schools where there was low attendance at the initial workshop, the trainer revisited the school at a more appropriate time, in order for the training to take place, and the maximum number of staff to attend [30]. The training workshops were seen as a key part of getting the momentum going and starting to change attitudes. In the “Us Girls” intervention [32], workshops were seen as successful in regards to sustainability and dissemination of the intervention. Conversely, a small number of participants in “Fit for Girls” [30] felt that the training could have been more specific to the issues faced in their schools, and more assistance was needed in relation to making changes at a school-based level.

**(b, c) Action plan, grant**

One intervention [30] required an action plan from each school. This detailed their goals and strategies for promoting physical activity among low active girls. Schools with approved action plans were provided with a start-up grant of £700.Two interventions mentioned financial grants [30],[31]. In the “Fit for Girls” intervention [30], this was seen more as an incentive to join and encouraged sustainability with existing resources as it could not be used to employ additional staff or coaches.

**(d) Partnership working**

Three studies of interventions mentioned partnership working, such as with community sports clubs and national governing bodies [30],[32],[33]. This involved forging relationships to ensure progressive and coherent pathways, which was seen as crucial for retention in team sport. All studies found that partnership work was helpful for increasing participation, attracting new participants, and enhancing resources. “Us Girls” [32] stated that initial recruitment of participants was a challenge. Partners could provide access to participants, use of facilities and equipment, and local knowledge of community needs and interests. Working with intact groups such as colleges, community groups, or places of work were found to be effective for recruiting participants to activity sessions. “Sportivate” [33] noted that this support aided sustainability of the intervention and was recorded as one of the top eight successes of the intervention.

**(e) Resources**

Two studies of interventions provided resources for their staff or volunteers [30],[32]. “Us Girls” [32] noted that the resources enhanced the delivery of the projects and was developed based on the process evaluation carried out in the early stage of the intervention. The resources included: on-line and hard copy resources such as: how-to guides, visits, and accessible telephone-based support. However, in the “Fit for Girls” intervention [30], there was a decrease in the number of PE staff that found the resources useful, from 88.9% in year 2 to 77% in year 3. The authors noted that this slight decrease could be a reflection of the time since they attended the training as, for many, it was two years since they had received training and the associated resources.

**(f, g, h) “How to” guides, gender-specific research and insights, merchandise**

“Us Girls” [32] was the only intervention to provide “How to” guides, gender-specific research and insights, and merchandise. The “How to” guides comprised of a series of 14 practical guides to help project managers, coaches and deliverers to increase and sustain young women's sport participation. However, there was no evaluation of effectiveness of the “How to” Guides or gender-specific research. In contrast, the merchandise was widely used by partners as incentives for recruitment and rewards for participation. Therefore, they were deemed effective as coaches participating in the intervention provided positive feedback on the use of incentives for promoting regular participation over the course of the intervention.

(3) Outcomes of the Interventions

**(a, b) Participation, retention**

All four studies [30]–[33] reported the outcome of participation and both “Sportivate” [33] and “Girls on the Move” [31] made reference to retention or sustained participation within team sport (see Table 3). Of those surveyed, 59.9% of PE staff said that “Fit for Girls” [30] had helped to increase girls' participation in PE, sport and physical activity. Specifically, 47.5% of PE staff said that the programme had helped increase participation among low active girls. “Us Girls” [32] successfully reached its target of engaging 30,000 young women in sporting activities. In “Girls on the Move” [31], it is estimated that around 1,800 girls took part in activities provided. Around one-half (53%) of girls had high attendance rates and were involved from the beginning to the end of their project. 25% of girls had low attendance at projects (attending less than one in four sessions), with some girls attending only one session before dropping out. On the other hand, “Sportivate” [33] reported that there had been a decrease in female participants in the programme from year 2-3 (-2.2%). Nevertheless, females were just as likely to be retained as males once they had found an activity which suited them.

**Table 3.** Outcomes of Interest

Name of Intervention	(a) Participation	(b) Retention	(c) Consultation with the adolescent girls	(d) Provision of clubs/sports	(e) Trying new sports	(f) Success of new clubs/sports	(g) Specific sporting preferences	(h) Motivating factors	(i) Perceived Barriers	(j) Importance of the Coach
Fit for Girls [30]	X		X	X	X	X	X	X	X	X
Us Girls [32]	X			X	X	X	X	X		X
Sportivate [33]	X	X		X		X	X			
Girls on the Move [31]	X	X		X			X			

Additionally, “Sportivate” [33] reported a significant drop in female participation amongst those aged 16–18 years. Although female participation was high for those aged 14 and 15, there was a −7.1% decrease in the level of female participation aged 16 when compared to participation levels aged 14, this was a similar decrease to year 2 (−7.4%). “Fit for Girls” [30] did not increase participation in extracurricular sport. 60.8% of girls reported not having taken part in an extra-curricular sport or physical activity during the previous week. This represents an increase from the previous year's evaluation, where just under half (47.5%) had not taken part. Although there was a decrease in the number of girls attending school sports clubs, there was an increase from 39% to 45% of girls participating in sports clubs outside of school, suggesting that the location and environment plays a role in participation.

**(c) Consultation with the adolescent girls**

Previous literature has suggested that consulting with the girls about specifics of what sports they would be interested in, and the format of delivery, could be a facilitator for participation. However, only “Fit for Girls” [30] referred to this, with 98.8% of PE staff saying that they had done this by year 3. Interviews with PE staff at the case study schools showed that they valued the consultation with girls and felt that it helped them, not only understand what it was girls wanted, but also helped with increasing participation levels.

**(d) Provision of clubs/sports**

All evaluations reported an outcome of increased provision of clubs/sports. PE staff from the “Fit for Girls” intervention [30] reported a total of 250 new clubs due to the influence of the intervention. “Us Girls” [32] successfully used taster sessions, festivals, and open days to give potential participants an idea of what the sessions would be like. It provided participants with a range of activities from competitive team sports to individual exercise-based sessions such as Zumba.

**(e) Trying new sports**

Two of the studies reported on the number of participants trying new sports [30],[32]. During the period of “Fit for Girls” [30], a large proportion of girls (62.2%) reported that they had taken part in an activity they had not tried before. In addition, half (49.2%) had taken part in girls-only activities. Although these activities cannot be directly attributed to “Fit for Girls” [30], the figures suggest that girls had access to a wide range of activities. Over a third (37.3%) had taken part in an activity outside of school as a result of trying it at school. This suggests that exposure to different physical activities within the school setting can have an impact on participation outside of school.

**(f) Success of new clubs/sports**

Three studies of interventions reported on the success of the new clubs/sports [30],[32],[33]. Girls only clubs had been a suggestion from previous literature, and was utilised in “Fit for Girls” [30]. Although many of the “disengaged” girls who took part in the focus groups felt more comfortable in single-sex PE groups, this was not necessarily a view shared by all of their peers. Furthermore, fewer girls agreed that boys and girls should do PE separately (30.8% compared with 37% over the course of the intervention). For many of the girls, being able to opt in to girls-only activities was really important in terms of their own confidence and enjoyment within the PE class and, consequently, their levels of participation. Less active girls were more likely to prefer girls only classes than more active girls (33.6% compared with 28.8%). “Us Girls” [32] connected the success of new activities with: effective organisation of sessions; marketing events using the strap-line fun, friends and fitness along with images of “normal” women; and using merchandise as incentives.

**(g) Specific sporting preferences**

All studies reported on specific sporting preferences of the participants [30]–[33]. “Fit for Girls” [30] reported no real changes in the preference of girls-only activities over the course of the intervention. In ranking order, from highest to lowest: dance, football, basketball, gymnastics, athletics, badminton, aerobics, hockey, netball and rounders ranked in the top 10. The only exception being rounders had replaced multigym in the top 10 from the previous year. The fact that dance, a non- team sport, was ranked first, suggests that the intervention had not increased the success of team sports. However, four out of the top ten were team sports, which is somewhat positive, but the intervention did not result in a preference for team sports. Relevant to this review, multi-sport sessions were very popular and participants enjoyed the chance to try different sports. Some “Us Girls” [32] participants expressed a preference for having fun, variety, and the social rewards of playing on a team without necessarily wanting to train for a particular sport. A number of participants stated that they enjoyed experiencing a variety of sports and activities rather than focusing on a particular sport.

Whilst variety was appreciated by a number of women, some of the younger women involved were committed to particular activities such as Streetdance or basketball and wanted to focus on improving their skill and working towards playing and performing to a high standard. It was concluded that offering multiple activities can be challenging for coaches as they need to access a range of equipment but can prove motivating to adolescent females who want to have fun and get fit, but are less interested in specialising in a particular sport. “Sportivate” [33] reported on the success of sports in relation to gender and retention. The evaluation found that there was a significant difference between the sports that retain the most male compared with those that retain female participants. The most successful sports in retaining men and boys were basketball 10.1% (n=2,340), followed by football 9.6% (n=2,208) and boxing, 6.5%, (n=1,505). For women and girls, the top three activities were gym/fitness 7.5% (n=1,119), followed by dance 7.1% (n=1,060), and boxing 6.9% (n=893). None of the female activities were team based sports, which could suggest that either: there was not a significant focus on team sports; or that team sport is not appropriate in retaining participation. This latter suggestion is not supported by current evidence as it has been suggested that some individual sports are up to 10.0% less likely to retain women and girls than team sports [33]. Therefore, despite these being the most delivered sports and activities in the ‘Sportivate’ intervention [33], they may not be the best at retaining women, perhaps contributing to the lack of progression against the female retain target. “Girls on the move” [31] tentatively found that team sports, such as basketball, football, hockey and netball were less popular with the girls. From the activities provided, it is clear that a broad range of activities appeal to girls and that no single activity will cater for all girls' needs.

**(h) Motivating factors**

Two of the evaluations referred to changes in motivating factors in regards to activity, over the course of the intervention [30],[32]. “Fit for Girls” [30] found that “being healthy” was the main motivation for being active, pre and post-intervention, although the proportion reporting this to be a motivator decreased from 71.5% to 61.8%. “Losing weight” and “looking better” both increased as motivating factors for adolescent girls post-intervention, being the second and fourth most common motivators respectively. These were the only two motivators which increased with age among girls. They also added that losing weight was not, by itself, sufficient motivation and that the enjoyable atmosphere of sessions was crucial to sustaining participation. This suggests that future development of sport interventions could consider including a body image component.

**(i) Perceived barriers**

“Fit for Girls” [30] reflected on the change in perceived barriers over the course of the intervention. It was found that, with the exception of “looking after family”, which showed no significant change, adolescent girls were more likely to report all other barriers to being physically active post-intervention. However, the two most common barriers remained consistent over time; these were “lack of skill” and “preferring to do other things with their time”. “Lack of time” significantly increased from 27.1% to 41.4% of participants reporting it as a barrier post-intervention. The proportion of adolescent girls reporting the cost of activities as a barrier also increased by 10% between the two time points. With the exception of helping to look after their family, “low active” girls were more likely than “more active” girls to report barriers to being physically active. The biggest differences related to preferring to do other things with their time, a lack of interest in physical activity and a perceived lack of skill (reported more by ‘low active’ participants).

**(j) Importance of the coach**

Two of the four evaluations report on the role of the coach in relation to participation in the activities [30],[32]. The feedback from the “Us Girls” [32] evaluation was that coaches played a crucial role in the success of the intervention and many participants highlighted the coach as a key factor in their enjoyment, motivation, and attendance. Desired qualities in a coach included: friendly, sense of humour, inspirational, and motivating. Many participants identified that they preferred a democratic, participatory style of coaching that allowed them to have a say in the sessions. Feedback from the “Fit for Girls” [30] focus group found that, for some, issues around weight and fitness were heightened by comments from a teacher in the PE class. For example, one group of girls referred to an incident where a male teacher had told them they were overweight and commented on their lack of fitness. Such negative comments may exacerbate girls' worries about their weight and body image. This style of teaching was clearly detrimental to girls' engagement, with the girls reporting this as a reason for opting out of PE.

## 4. Discussion

This is the first systematic review to examine evidence for the effectiveness of team sport interventions aimed at secondary school aged girls (aged 11–18 years) on team sport participation and wider physical activity outcomes. Three of the four interventions assessed were girls-only interventions [30]–[32] whereas Sportivate [33] was mixed-gender. In order to increase participation, authors utilised numerous health promoting approaches to elicit change: training for existing staff; action plan documenting organiser's strategy; start-up grants; partnership working to provide access to participants, use of facilities and equipment, and local knowledge of community needs and interests; resources, “How to” guides, gender-specific research and insights to aid staff and coaches in developing and delivering their intervention; merchandise as an incentive to participants. Outcomes that were reported on and discussed included: participation and retention in team sports; the positives of making the adolescent girls feel involved by consulting with them in the development of the intervention; increasing provision of clubs/sports resulted in the participants trying new sports; the specific sporting preferences of the participants.

With regard to impact of the interventions on girls' wider physical activity outcomes, study authors identified barriers to physical activity change such as perceived lack of skill and a preference for doing other things with their time. Maintaining a healthy lifestyle was recognised as an important motivating factor for the adolescent girls, and the importance of the coach in creating a fun and encouraging atmosphere was also discussed. This finding highlights the potential importance of sporting role models. For example, if women's team sport was given a higher platform, it could begin to inspire young people to initiate and maintain sports participation, and begin to change the social norm around what it is for women to be “sporty”. If promoted in the correct way, the success of women in team sport in recent times could be built on to create a “festival effect”, a phrase derived from the promotion of the London 2012 Olympic Games as a national event [34]. In the last two years alone, England women have brought home bronze medals in both the football and netball world cups, and Olympic gold medals in hockey. This success could be promoted in a way to encourage others to play team sport.

### 4.1. Strengths and Limitations

A limitation of the review is that none of the final studies had gone through a formal peer review process as they were all obtained from grey literature, mainly national sports council websites (e.g. Sport England). Part of the peer-review process ensures that published studies address and minimise bias, discuss and explain the lack of randomisation of participants to intervention or control. This ensures that the study design and analysis are rigorous and conclusions are justified. Despite physical inactivity becoming a priority in recent years, there is a lack of high quality evaluations of any physical activity interventions, especially UK based and in particular focussing on team sport. Possible reasons for this include a lack of available funding for evaluations of physical activity interventions which may mean that the evaluation is scaled down to such a level that methods do not meet the standards required for peer-reviewed publication [35]. Alternatively, the group undertaking the evaluation could be more interested in publishing reports that are of a more useful and user-friendly format to read for the end user, which are likely to be non-academics such as teachers, local government reps, policy makers [36]. This could explain why none of the evaluations found through grey literature searches had made it to the database systems yet and would explain why there was a lack of methodological description within the papers, as it is the findings or recommendations that the market audience is interested in and not the methodology behind the findings. These insights are a major strength of this study and emphasise how grey literature cannot be overlooked, especially in the context of evaluations of public health interventions.

It should be noted that, although a systematic review aims to incorporate the findings of all relevant literature, it is probable that there are some small-scale locally-based interventions that were not found through the searches as no ‘gold standard’ method for rigorous systematic grey literature searching exists [37]. Additionally, there is the potential of publication bias, as national sports councils are unlikely to publish results of interventions that were not found to have at least minimal positive effect [37].

### 4.2. What is Already Known and What This Study Adds

To support an increase in local physical activity, the Department of Health are promoting tools such as: the EAED (Everybody Active, Every Day) toolkit, walking cities and new physical activity initiatives [38] but there is no known nationally promoted tool for team sport participation. Studies in this systematic review were limited to those based in the UK, as social and cultural expectations about sport and physical activity are known to be very different between countries[39] and the intention was to identify implications for UK interventions and policy. However, across many nations, including Denmark, Sweden, Finland, Australia, New Zealand and the Netherlands there is a, policy-driven, realisation that sport can play an important role in achieving community health objectives [16].

Future development of UK interventions could be informed by the approaches taken in this systematic review, such as action planning of the proposed strategies, working and building relationships with existing organisations, providing merchandise as an incentive for adolescent girls to participate in team sports. This review also highlights the importance of reporting on crucial factors, such as which sports are most effective in sustaining participation over time. The whole process of design, implementation, analysis and dissemination should be thoroughly investigated and explore in the planning stage to ensure that the relevant data is collected from the beginning. The recommendations of this systematic review could be used to develop and evaluate future interventions and policies.

## 5. Conclusion

This review presented a narrative assessment of four studies of physical activity interventions, with a specific focus on secondary school aged girls' participation in team sports and other related health outcomes. The findings of the review suggest that further research should focus on filling the many gaps identified, such as: the need for the evaluation of rigorous, high quality interventions designed to promote team sport among adolescent girls.

The results of this review identify recommendations for future interventions. For example, the importance of consulting with the girls; the importance of encouraging girls to try new sports and sustain participation; the need for relatable, healthy role models in the media; and the role of the coach, were all identified as areas for consideration.

To move forward, there is a need for the provision of quality sport participation data to supply the evidence to inform well-structured programmes and policies to meet the community needs. This is not possible if sport participation remains hidden in the broader physical activity context, or is not investigated in more detail.

## Figures and Tables

**Supplementary 1:** Search strategy used to identify potentially relevant studies from online databases.

#	Query	Limiters/Expanders	Results
S1	girl* or school girl* or female* or young wom* or adolescen* or teen* or child* or pre-adolescen* or pre-teen* or high school* or “year 7 or 8”	Search modes - Boolean/Phrase	11,414,315
S2	physical activit* or exercis* or sport* or game* or basketball* or volleyball* or water polo* or handball* or lacrosse* or cricket* or football* or netball* or hockey* or team sport* or team ball sport* or team game*	Search modes - Boolean/Phrase	1,920,656
S3	intervention* or campaign* or strateg* or promot* or program* or initiativ*	Search modes - Boolean/Phrase	4,677,670
S4	increase*	Search modes - Boolean/Phrase	4,607,284
S5	participat*	Search modes - Boolean/Phrase	828,697
S6	S4 N3 S5	Search modes - Boolean/Phrase	10,748
S7	S1 AND S2 AND S3 AND S6	Search modes - Boolean/Phrase	870
S8	S1 AND S2 AND S3 AND S6	Limiters - Published Date: 20050101-20151231 Search modes - Boolean/Phrase	645
S9	S2 AND S6	Search modes - Boolean/Phrase	2,591
S10	S2 N3 S6	Search modes - Boolean/Phrase	1,884
S11	S1 AND S3 AND S10	Search modes - Boolean/Phrase	683
S12	S1 AND S3 AND S10	Limiters - Published Date: 20050101-20151231 Search modes - Boolean/Phrase	510
S13	S1 AND S3 AND S10	Limiters - Published Date: 20050101-20151231 Narrow by Subject Age0: - adolescent: 13-18 years Search modes - Boolean/Phrase	110
S14	S1 AND S3 AND S10	Limiters - Published Date: 20050101-20151231 Narrow by Subject Age0: - all child: 0-18 years Search modes - Boolean/Phrase	107
S15	S13 OR S14	Search modes - Boolean/Phrase	200

**Supplementary 2:** Search History of Grey Literature

Website	Intervention/Campaign	Report/Evaluation Found?
http://www.youthsporttrust.org/	Change 4 Life sports club	
	Fit for Girls	**Yes**
	Hubs school	
	Junior Club Champions	
	Sainsbury's School Games	
	School Sport Clubs	
	Skills 2 Achieve	
	Sky Sports Living for Sport	
	Sporting Promise- TOP Sport	
	Sporting Promise- yoUR Activity	
	TOP Sportsability	
	Young Ambassadors	
	YA Peer Leaders	
	Innovation Schools	
	Girls Active	**Yes**

https://www.sportengland.org/	This Girl Can	
	Go Where Women Are	
	I Will if You Will	
	Women in Sport	
	Back to Netball	
	Us Girls	**Yes**
	Sportivate	**Yes**

http://www.sportscotland.org.uk/	Active Schools	
	Active Girls	

http://sport.wales/		

http://www.youthscotland.org.uk/	Girls on the Move	**Yes**

https://www.womeninsport.org/	Changing the Game for Girls	Yes* also found through database search

**Supplementary 3:** Critical Appraisal Template

**Study identification:** (Include full citation details)		
**Study design:** Which experimental and observational study designs best describe the paper's underpinning study design		
**Assessed by:** (Name and date)		

**++** All or most of the checklist criteria have been fulfilled, where they have not been fulfilled the conclusions are very unlikely to alter.**+** Some of the checklist criteria have been fulfilled, where they have not been fulfilled, or not adequately described, the conclusions are unlikely to alter.**−** Few or no checklist criteria have been fulfilled and the conclusions are likely or very likely to alter.**NA** should be reserved for those study design aspects that are not applicable given the study design under review		

**Section 1. Population and Recruitment:**		**Comments:**
1.1 Is the population of the study well defined? Consider: Was the setting (secondary schools, community centres etc.), location (urban, rural), population demographics etc. adequately described?	**++** **+** **−** **NA**	
1.2 Was the recruitment strategy defined and appropriate to the aims of the study?	**++** **+** **−** **NA**	

**Section 2. Intervention:**		**Comments:**
2.1 Was there a clear statement of the aims of the research?	**++** **+** **−** **NA**	
2.2 Was the intervention well described? Was the development, process and length of intervention adequately described?	**++** **+** **−** **NA**	
2.3 Is there a statement referring to compliance with the intervention?	**++** **+** **−** **NA**	

**Section 3. Outcomes:**		**Comments:**
3.1 Was there a satisfactory statement of criteria for outcome measures?	**++** **+** **−** **NA**	

**Section 4. Data Collection:**		**Comments:**
4.1 Was the data collected in a way that addressed the research question? Consider: If the setting for data collection was justified If it is clear how data were collected (e.g. focus group, semi-structured interview etc.) If the researcher has justified the methods chosen If the researcher has made the methods explicit (e.g. for interview method, is there an indication of how interviews were conducted, or did they use a topic guide)? If methods were modified during the study. If so, has the researcher explained how and why? If the form of data is clear (e.g. tape recordings, video material, notes etc) If the researcher has discussed saturation of data	**+++−NA**	

**Section 5. Analysis**		**Comments:**
5.1. For quantitative analysis, was there a statement adequately describing or referencing all statistical methods? Consider: Was Intention to treat (ITT) analysis conducted? Were all participants (including those that dropped out or did not fully complete the intervention course) analysed or referenced to?	**++** **+** **−** **NA**	
5.2 For qualitative analysis, was the data analysis sufficiently vigorous? Consider: If there is an in-depth description of the analysis process If thematic analysis is used. If so, is it clear how the categories/themes were derived from the data? Whether the researcher explains how the data presented were selected from the original sample to demonstrate the analysis process If sufficient data are presented to support the findings To what extent contradictory data are taken into account Whether the researcher critically examined their own role, potential bias and influence during analysis and selection of data for presentation	**++** **+** **−** **NA**	

**Section 6. Conclusions**		**Comments:**
6.1 Were the conclusions drawn from the analysis justified? Consider: Is there adequate discussion of the evidence both for and against the researcher's arguments? Has the researcher has discussed the credibility of their findings (e.g. triangulation, respondent validation, more than one analyst) Were there significant flaws in the study design?	**++** **+** **−** **NA**	
6.2 Are the findings discussed in relation to the original research question?	**++** **+** **−** **NA**	

**Supplementary 4:** Data Extraction Template

**Study Details**	
**Full Reference**	
**Source through which found**	
**Population Details**	
**Number of Participants**	
**Gender**	
**Country**	
**How recruited**	
**Full Details of the Intervention**	

**All Study Outcomes**	

**Relevant Results**	


**Author's Conclusions**	

**What worked?**	

**What didn't work**	

**Relevant Notes from Critical Appraisal**	

**Recommendations for the Future**	

**Additional Comments**	

